# An automatic entropy method to efficiently mask histology whole-slide images

**DOI:** 10.1038/s41598-023-29638-1

**Published:** 2023-03-15

**Authors:** Yipei Song, Francesco Cisternino, Joost M. Mekke, Gert J. de Borst, Dominique P. V. de Kleijn, Gerard Pasterkamp, Aryan Vink, Craig A. Glastonbury, Sander W. van der Laan, Clint L. Miller

**Affiliations:** 1 Department of Computer Engineering, University of Virginia, Charlottesville, VA 22908 USA; 2 Center for Public Health Genomics, University of Virginia, MSB 3231, PO Box 800717, Charlottesville, VA 22908 USA; 3 Human Technopole, Viale Rita Levi-Montalcini, 1, 20157 Milano, Italy; 4 Department of Vascular Surgery, Division Surgical Specialties, University Medical Center Utrecht, Utrecht University, Utrecht, The Netherlands; 5 Central Diagnostics Laboratory, Division Laboratories, Pharmacy, and Biomedical Genetics, University Medical Center Utrecht, Utrecht University, Heidelberglaan 100, 3584 CX Utrecht, The Netherlands; 6 Department of Pathology, Division Laboratories, Pharmacy, and Biomedical Genetics, University Medical Center Utrecht, Utrecht University, Utrecht, The Netherlands; 7 Department of Public Health Sciences, Department of Biochemistry and Molecular Genetics, University of Virginia, Charlottesville, VA 22908 USA

**Keywords:** Translational research, Image processing, Machine learning, Diseases

## Abstract

Tissue segmentation of histology whole-slide images (WSI) remains a critical task in automated digital pathology workflows for both accurate disease diagnosis and deep phenotyping for research purposes. This is especially challenging when the tissue structure of biospecimens is relatively porous and heterogeneous, such as for atherosclerotic plaques. In this study, we developed a unique approach called ‘EntropyMasker’ based on image entropy to tackle the fore- and background segmentation (masking) task in histology WSI. We evaluated our method on 97 high-resolution WSI of human carotid atherosclerotic plaques in the Athero-Express Biobank Study, constituting hematoxylin and eosin and 8 other staining types. Using multiple benchmarking metrics, we compared our method with four widely used segmentation methods: Otsu’s method, Adaptive mean, Adaptive Gaussian and slideMask and observed that our method had the highest sensitivity and Jaccard similarity index. We envision **EntropyMasker** to fill an important gap in WSI preprocessing, machine learning image analysis pipelines, and enable disease phenotyping beyond the field of atherosclerosis.

## Introduction

Atherosclerosis is a chronic inflammatory process resulting in arterial stiffening and plaque formation, and is the leading cause of myocardial infarction, ischemic stroke, and peripheral artery disease^1,2^. Historically, researchers and pathologists have characterized atherosclerotic plaque through standard histology and light microscopy analysis^3–6^. The composition of atherosclerotic plaques is highly variable, with different plaque types having distinct clinical manifestations^3,7^. For instance, more stable, fibrous-rich atheroma plaques are typically asymptomatic, whereas unstable, thin-cap fibroatheroma plaques are more prone to rupture and thrombus formation underlying cerebral or coronary events^5,6^. However, the value of atherosclerotic plaque composition in predicting cardiovascular events remains a subject of debate and ongoing research^7–9^.

Histological analysis of atherosclerosis is mainly used in the research setting and can reveal the extent of disease progression as well as underlying etiology. For example, in pre-clinical models of atherosclerosis, the amount of smooth muscle content and collagen extracellular matrix in the plaque often correlates with greater plaque stability^8^. These features can now be easily captured from stained tissue sections and digitized as high-resolution whole-slide images (WSI). WSI data provides a rich resource for quantitative and qualitative image analysis and has been a focus of digital pathology^10^. Overall, this has simplified archiving, enabled remote diagnosis, and accelerated both clinical decision-making and research investigations^11^.

Accurately identifying tissue (foreground) from background is a necessary initial step in WSI analysis in digital pathology workflows. However, the process of WSI acquisition has its own technical challenges. It is often challenging to identify or "mask" the entire non-tissue region from WSI, e.g., due to factors such as dust, translucent tissue, or weak immunostaining, which may lead to incorrect diagnosis^12^. Since rescanning and manually inspecting slides is labor-intensive, automated algorithms that highlight the tissue-specific areas to be analyzed is a more efficient solution^13^. These algorithms also adjust for inter- and intra-image aberrations in color, staining and slide preparation through more robust tissue segmentation.

The most straightforward method for identifying tissue on a white background is to set a specified threshold for the grayscale version of the image^14^, specific color channels^15^, or the optical density of the red, green, and blue (RGB) color channels^16^. Alternatively, tissue can be identified from WSIs split into a uniform patch grid that is subjected to grayscale image threshold^17,18^. Otsu's thresholding^19^ is a commonly used tissue masking method in WSI analysis studies^13,20,21^. Several image segmentation methods have been developed to extend Otsu’s method and improve foreground extraction from WSI, such as GrabCut^22^ and Foreground Extraction from Structural Information (FESI)^23^. While these methods are easy to implement, it has been difficult to generalize to more variable stains or clinical specimens. A more accurate approach is still needed in digital pathology workflows, especially for specimens containing small empty spaces between extracellular matrix fibers (e.g., collagen) or due to decalcifying procedures, and transparent tissue (e.g., adipose or lipid droplets) in atherosclerotic plaques. Image thresholding techniques based on entropy maximization have been routinely applied to infrared image analysis^24–26^. Similar to infrared images with poor contrast and noise, WSI may also benefit from entropy thresholding to distinguish tissues from the slide background; to our knowledge this approach has not yet been applied to WSI.

In this paper, we propose a fully automated approach for separating foreground (tissue) and background in WSI of HE-stained atherosclerotic plaque samples. Our method, **EntropyMasker**, is unaffected by changes in scanning or image processing conditions, by using a measure of local entropy and generating corresponding binary tissue masks. This allows for increased scalability across various tissue contexts and implementation in both machine learning and computer vision cell segmentation pipelines, e.g., using CellProfiler^27^ and slideToolKit^28^.

## Methods

### Our method: EntropyMasker

For WSI processing, ideally the slide background is evenly achromatic, with the background mostly one color, e.g., black (fluorescence) or white (brightfield). Unfortunately, in practice, there is often inconsistency of the background color due to variations in image capturing color temperature (e.g., from shades of blue to yellow), resulting in edge artifacts from tile-based scanning methods^29^. Therefore, the slide background cannot be easily masked by transforming the images from RGB color space to the HSV (Hue-Saturation-Value) and excluding the white color (0°, 0%, 100%).

In information theory^30^, entropy is defined as the log-base-2 of the number of possible outcomes for a sent/received message. In image analysis, entropy refers to the degree of randomness or complexity of pixels in a defined region or neighborhood of pixels. We defined the local entropy of a specific region as follows:

$$H\left( F \right) = - \mathop \sum \limits_{i = 0}^{255} p_{F} \left( i \right)log_{2} p_{F} \left( i \right),$$

where $p_{F} \left( i \right)$ represents the probability of a grayscale pixel, $i$, in a local footprint region.

In the case of $p_{F} \left( i \right) = 0$ for some $i$, the corresponding sum value and $0log_{2} 0$ is set to $0$, which is consistent with the limit: $\mathop {lim}\limits_{{p_{F} \to 0^{ + } }} p_{F} \log \left( {p_{F} } \right) = 0$.

Based on this approach, homogeneous pixels within local neighborhood regions (disk-shaped footprints with radius of 5) have a low probability and contribute less to the local entropy, whereas heterogeneous pixels within local neighborhood regions have a high probability and contribute more to the local entropy. Also, the background of an image has lower entropy due to higher brightness and more homogeneous texture, whereas foreground tissue has higher entropy due to the diversity of pixel intensities in the local neighborhood. In other words, the heterogenous pixels in tissue with high entropy are more informative than homogenous background pixels with low entropy^31^.

The purpose is to extract the tissue (foreground) from the background. We first calculated local entropies and then we made a histogram of all local entropies. Since the background of the image has a lower entropy value, we first defined the threshold as the local minima of the histogram of the entropy map. Next, we applied this threshold to convert the local entropy map into a binary map which distinguishes background noise from tissue (foreground) in the WSI.

### Otsu’s method

Otsu's method is a clustering-based image thresholding algorithm^19^, commonly utilized in image analysis and digital histopathology applications^20,29,32–34^. Following a bimodal histogram, the technique assumes that the image has two classes of pixels (tissue pixels and background pixels). It determines the best threshold for dividing the two groups such that their total intra-class variance is as low as possible.

### Adaptive thresholding

Adaptive thresholding is another widely used method for calculating the threshold value for smaller areas of WSIs^35–38^. Typically, these threshold values are calculated in two different ways, either using the mean of the neighborhood area, or a Gaussian weighted sum of neighborhood values.

### slideMask method from slideToolKit

The slideToolKit^28^ is an assistive tool set for the histological quantification of whole-slide images (https://github.com/swvanderlaan/slideToolKit). The slideMask tool in slideToolKit uses convert from ImageMagick (https://imagemagick.org) and a miniature version of the WSI to generate a mask (https://github.com/swvanderlaan/slideToolKit/blob/master/slideMask). The image is first blurred to eliminate dust and speckles. Then Fuzz in ImageMagick^39^ (which is computed as the root mean squared difference between two colors of the pixels) is used to create a fuzzy, non-stringent selection to identify the white background, which is subsequently substituted with black. The slideMask tool has settings for blur and fuzziness that may be explored and altered. Manual adjustments may be made to generated masks in any image editor (such as free GNU Image Manipulation Program; GIMP^38,40^). Unwanted regions on the WSI (such as marker stripes or air bubbles beneath the coverslip) may necessitate this procedure.

All proposed methods were implemented in the Python programming language using OpenCV^41^ and other required packages (see Data and code availability for more details).

### Athero-express biobank study

#### Patient population

Atherosclerotic plaques were obtained from patients undergoing an arterial endarterectomy procedure and included in the Athero-Express Biobank Study (AE), an ongoing biobank study at the University Medical Centre Utrecht (Utrecht, The Netherlands) and the St. Antonius Hospital (Nieuwegein, The Netherlands)^3^. The medical ethical committees of the respective hospitals approved the AE which was registered under number TME/C-01.18. This study complies with the Declaration of Helsinki, and all participants provided informed consent. However, considering national laws plaque-material are considered ‘waste biomaterial’ and are always allowed to be used without any personal information regardless of informed consent. In this study we also considered this ‘waste biomaterial’, hence no clinical information, e.g., age, is given here nor when sharing data of the relevant samples (n = 3). The study design of the AE was described before^3^, but in brief: during surgery blood and plaques are obtained, stored at − 80 °C and plaque material is routinely used for histological analysis^3,42^.

#### Whole-slide staining and scanning

The standardized (immuno)histochemical analysis protocols used in the AE biobank have been described previously^3,42^. In short, 4‐micron cross‐sections of the paraffin‐embedded segments were cut using a microtome, and 8 different stains were applied for endothelial cells (CD34), macrophages (CD68), elastic Van Gieson (EvG), fibrin, red blood cells (glycophorin C, GLYCC), hematoxylin and eosin (HE) for nuclei, collagen (picrosirius red, SR), and smooth muscle cells (SMCs, α‐actin) on consecutive slides. We set up ExpressScan to obtain whole-slide images (WSIs) by scanning stained slides at 40× using a Roche Ventana iScan HT (https://diagnostics.roche.com/global/en/products/instruments/ventana-iscan-ht.html) or Hamamatsu C12000-22 Digital slide scanner (https://www.hamamatsu.com/eu/en/product/life-science-and-medical-systems/digital-slide-scanner/index.html). WSIs were stored digitally as z-stacked .TIF (Roche) at 0.25 micron/pixel or .ndpi (Hamamatsu) at 0.23 micron/pixel brightfield microscopy images^10^. The slides numbers used for this project are listed in Supplemental Table 1.

**Table 1:** Comparison of different masking methods.

Method	Jaccard index	sensitivity	False positive rate	Pixel accuracy
SlideMask	0.5273 ± 0.29	0.5451 ± 0.30	0.0153 ± 0.06	0.9133 ± 0.08
Otsu’s	0.5499 ± 0.25	0.5608 ± 0.25	0.021 ± 0.09	0.8994 ± 0.10
Adaptive_mean	0.7322 ± 0.21	0.7691 ± 0.24	0.0278 ± 0.06	0.9452 ± 0.06
Adaptive_ gaussian	0.7702 ± 0.20	0..8597 ± 0.18	0.0393 ± 0.09	0.9485 ± 0.07
**EntropyMasker**	**0.8837 ± 0.08**	**0.9685 ± 0.06**	**0.0176 ± 0.02**	**0.9111 ± 0.07**

The method with the highest evaluation metric is shown in bold. Metric values represent mean ± SD.

#### Manual tissue annotation and masking

The ground truth tissue areas for each WSI were annotated manually at 40 × magnification using QuPath^43^. If the tissue had disintegrated into several disjointed fragments during preparation or staining the annotators marked them with a single enclosing polygon. Binary masks of these tissue annotations were then generated using groovy scripts in QuPath.

### Statistical analyses

We assessed the algorithms using several metrics: The Jaccard index, sensitivity, False positive Rate (FPR) and pixel accuracy, comparing the algorithms' output to the ground truth binary reference masks of the 97 images in the test set.

Jaccard index was applied to assess the performance of our masking algorithms, by measuring the overlap area of target masks with the ground truth masks annotated by experts and masks of the algorithm outputs divided by the area of the union of both masks. Mathematically, sensitivity, false positive rate and pixel accuracy can be expressed as:

$$Sensitivity = \frac{TP}{{ TP + FN}},$$

$$False positive rate = { }\frac{FP}{{FP + TN}} ,$$

$$Pixel accuracy = \frac{TP + TN}{{TP + FP + FN + TN}}.$$

### Evaluation and results

**Figure 1:**
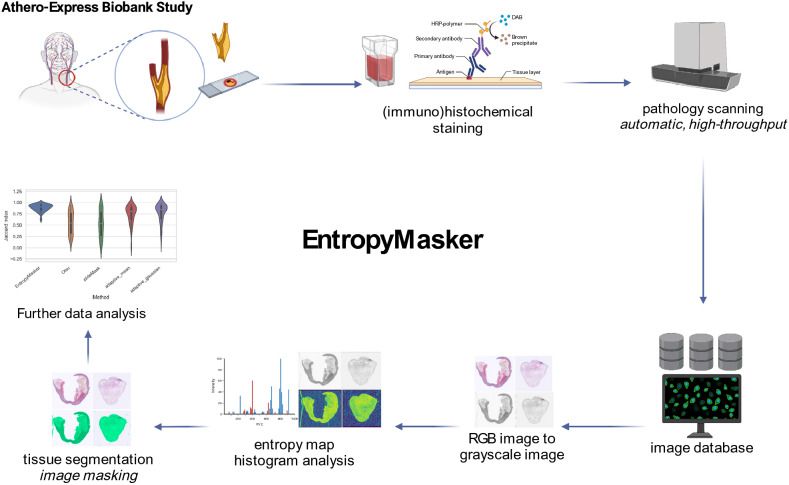
Schematic overview of the EntropyMasker method for masking whole-slide images. Atherosclerotic plaque tissue samples were collected from arterial endarterectomy surgeries, sectioned and mounted on tissue slides as part of the Athero-Express Biobank Study. The slides were subjected to histological staining for overall morphology (hematoxylin & eosin; HE), collagen deposition (Picrosirius red; SR), fibrin deposition (Fibrin), elastin fibers (Elastica Van Gieson; EVG) or immunohistochemical staining for cell-type specific markers: alpha-smooth muscle actin (a-SMA), macrophages (CD68), endothelial cells (CD34), or erythrocytes (glycophorin C). Whole-slide images of each slide were generated using high-resolution scanners and images were stored in a database (~ 25,000 images). Red, Green, Blue (RGB) color images were converted to grayscale images and grayscale pixels were used to calculate entropy threshold map for histogram analysis, and tissue segmentation was performed to create tissue mask of WSIs for subsequent data analysis and benchmarking. Schematic was created with BioRender.com.

We developed **EntropyMasker**, a novel masking method using entropy-filtering (Fig. 1). First, we compared our method against three conventional thresholding methods: Otsu’s, adaptive thresholding using the mean of neighborhood area as the threshold, and adaptive thresholding using the Gaussian weighted sum of neighborhood values as the threshold. We also evaluated these methods against the previously developed slideMask tissue masking method in the slideToolKit^28^.

Application of quantitative WSI analysis pipelines requires access to uniformly processed and well-characterized tissue biobanks. The Athero-Express study^3^ is a large-scale vascular tissue biobank comprising over 3600 carotid and femoral endarterectomy surgical specimens, which include detailed clinical outcomes and follow-up. This has led to several prospective studies to correlate local atherosclerotic plaque composition with future local and systemic vascular outcomes, using histology^7–10^, RNA^44,45^, genetics^46^, and protein^47,48^ data. In the histology-based analyses, local plaque indices such as plaque hemorrhage and neovascularity were shown to correlate with more adverse vascular outcomes^7,9^. We evaluated each method using 97 randomly selected WSIs from Athero-Express (Supplemental Table 1 for a minimalistic baseline table and Supplemental Table 2 for details on usage) at 40X magnification on layer 6 (pixel spacing at layer 6 is around 8.36 µm) consisting of 8 different stains: HE, picrosirius red, Fibrin, EVG, a-SMA, CD34, CD68, and GLYCC. We did not apply our method to layer 1 due to memory constraints.

**Figure 2:**
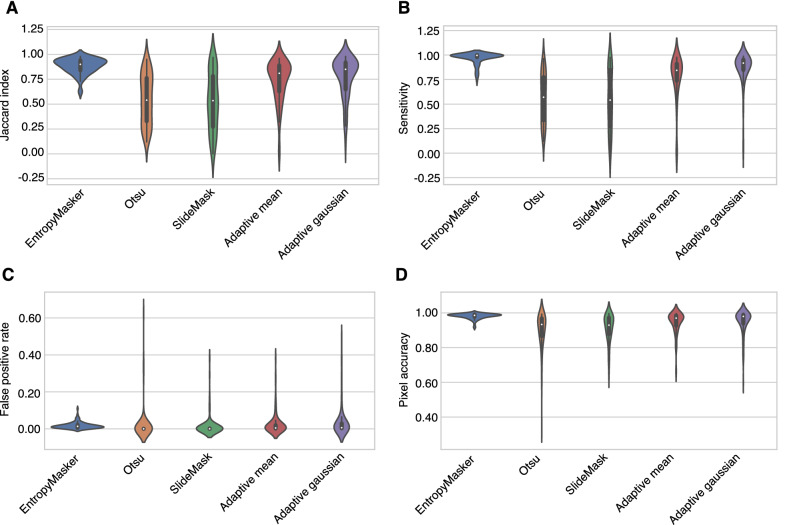
Evaluation metrics for different masking methods. Comparison of **EntropyMasker** with other masking methods: Otsu, slideMask, and adaptive thresholding methods, using manually annotated tissue sections as ground truth. 97 images from 59 individual patient samples were randomly selected and used to evaluate each method by calculating the (**A**) Jaccard index (intersection over union metric), (**B**) sensitivity, (**C**) false positive rate, and (**D**) pixel accuracy.

The average Jaccard index, also referred to as the Intersection over Union (IoU) metric, of the adaptive thresholding method where its threshold value is the weighted sum of neighborhood values where weights are a gaussian window was 0.7702 and the average sensitivity was 0.8597 (Table 1, Supplemental Table 3 includes results for all stains and methods). This masking method produced the highest pixel accuracy among all the traditional methods which was 0.9485. Otsu’s method had a much lower false positive rate than any of the approaches examined at the cost of much lower average Dice scores and sensitivity levels. The quality of the outcome of the slideMask algorithm was unevenly distributed resulting in a relatively low Jaccard index (IoU) across all the tested methods (Fig. 2). The average false positive rate was 0.0153, which is the lowest of all masking methods in comparison, and its average pixel accuracy was 0.9133. Our automatic local entropy-based masking method, **EntropyMasker**, had the best average Jaccard index (IoU) 0.8837 and the highest sensitivity 0.9685, outperforming Otsu’s method, two adaptive methods and slideMask masking method in both metrics. The average false positive rate was 0.0176 which is much lower than Otsu’s method, and two adaptive methods.

**Figure 3:**
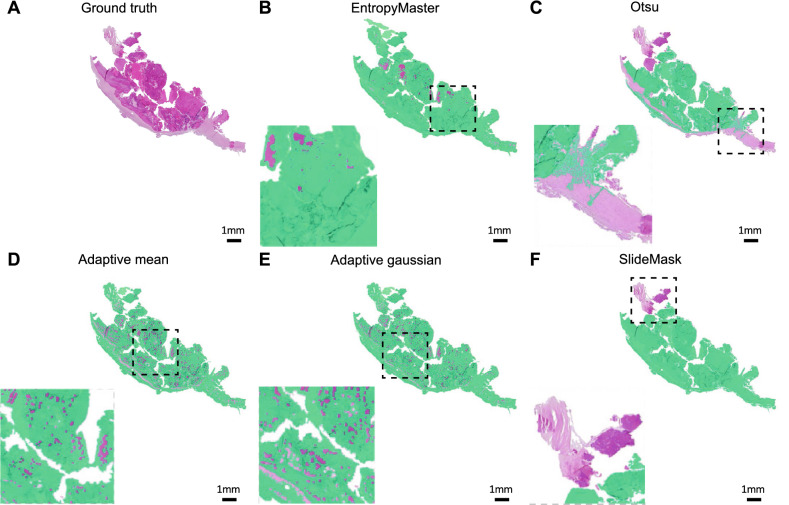
Comparison of results of 5 masking methods for a randomly selected H&E-stained whole-slide image. (**A**) Expert generated annotation (ground truth) using Qupath Software (Version 0.3.2)^43^. (**B**) Our EntropyMasker method. (**C**) Otsu’s method. (**D**) Adaptive method where the threshold value is the mean of neighborhood area. (**E**) Adaptive method where the threshold value is the weighted sum of neighborhood values where weights are a gaussian window. (**F**) slideMask method. All the generated tissue masks are overlaid by pseudocolor (green).

**Figure 4:**
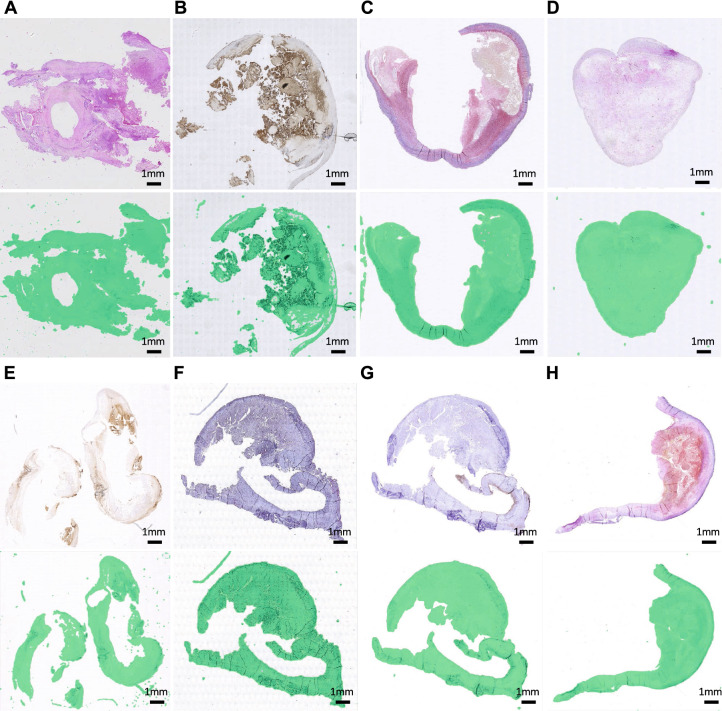
Generalizability across the different antibody-specific or histology stains. Images of the upper part are the original whole-slide images at layer 6 with 8 different types of staining methods respectively and images of the lower part are the examples of masking results of our EntropyMasker method where generated tissue masks are overlaid with pseudocolor (green). (**A**) H&E; (**B**) Glycophorin C; (**C**) Sirius red; (**D**) Fibrin; (**E**) CD68; (**F**) CD34; (**G**) Smooth muscle alpha-actin; (**H**) EVG.

The results of comparing the 5 masking methods for an H&E-stained whole-slide image are shown in Fig. 3 where generated tissue masks are overlaid by pseudocolor (green). To show the generalizability of our automatic entropy method, we tested on whole-slide images with 8 different types of staining. Examples of masking results of our proposed method are shown on Fig. 4 where generated tissue masks are overlaid by pseudocolor (green).

Since we used two different scanners, we also evaluated the **EntropyMasker** performance using different image formats (.ndpi or .TIF), which did not influence the Jaccard index (Supplemental Fig. 1). Also, to mitigate the influence of the porosity of the tissue, we evaluated these methods on both high (20X) and low (5x) resolution WSIs, however the performance was unaffected (Supplemental Table 4).

The computation speed for Otsu’s method, adaptive methods, slideMask and proposed **EntropyMasker** method on a 3.6 GHz Quad-Core Intel Core i7 processor, 32 GB 2400 MHz DDR4 memory iMac are 0.08, 0.11, 0.25 and 0.34 s per one million pixels, respectively.

## Conclusions and discussion

Overall, our automatic entropy masking method, **EntropyMasker**, performed well on atherosclerotic plaque cross-sectional WSIs derived from 8 different types of stains. With a relatively high average Jaccard index our method was able to separate out the foreground and background accurately and consistently from these complex images (Fig. 2 and 4).

In comparison, we observed the popular masking method, Otsu’s, and our previous method, slideMask, both tended to miss some tissue areas along the tissue boundary. This likely results from the relatively lower intensity of these areas, where Otsu’s method sets a threshold in the middle of two peaks, thus partially omitting the higher intensity pixels compared to the threshold (Fig. 2). The slideMask method computes the root mean squared difference between two colors, however this becomes problematic when the intensity of tissue area pixels is relatively large (Fig. 2).

We also found that the adaptive methods tend to produce ‘porosity’ during the masking, since these methods miss small regions of the image which are supposed to be regarded as tissue (Fig. 2). This large scale missing tissue makes Otsu’s, slideMask and adaptive thresholding methods impractical for use in many image processing workflows^10,28^, especially when applied to atherosclerotic plaque images. Given the limited availability and heterogeneous nature of these plaques, an ideal masking method will retain all the plaque components, which can be used for automatic cellular segmentation tasks to discover tissue or compartment-specific markers for disease progression and phenotyping. This is also a valuable feature of **EntropyMasker**, which can be implemented at the preprocessing stage of various machine learning pipelines for other diseases and research areas which require a complete WSI masking step^49–51^.

Despite the accurate and consistent performance of **EntropyMasker**, it is worth pointing out some known limitations. In comparison to the other methods that we evaluated, our method has the highest sensitivity, and thus tends to over-include a few pixels outside the edges of tissue (Fig. 3). While these regions represent a small fraction of the total tissue areas that we tested, this should be more carefully considered when evaluating smaller tissue sections^11^. Also, our method had slightly lower pixel accuracy than the adaptive methods, which may be important for tasks involving more subtle intensity differences in defining target regions of the tissue^52^. Compared to the other methods discussed, ours is slightly slower (0.34 s per one million pixels compared to 0.11 s for Otsu’s) which may pose an issue when examining thousands of images, although this should be negligible since our method is scalable on any high-performance compute cluster.

In conclusion, we demonstrated the effectiveness of our proposed method for tissue masking on human atherosclerotic plaque WSIs of different types of stains including HE, CD34, CD68, EVG, smooth muscle cell α‐actin, picrosirius red, fibrin and Glycophorin C. By evaluating our method against other popular masking methods and those recently developed, we also demonstrated that our entropy-based masking method is scalable and had optimal performance across these WSIs. Given its scalability in WSI processing, we envision **EntropyMasker** to be readily adopted across various disease contexts and implemented in existing pipelines as an input to both deep learning and cell counting tasks.

## Supplementary Information

Supplementary Information.

## Data Availability

The full histological data used in this study are available here https://doi.org/10.34894/GN4YOS including the high- and low-resolution whole-slide images. All documented code and the tutorial to run **EntropyMasker** can be found here https://github.com/CirculatoryHealth/EntropyMasker.
