# Supplementary material for: An automatic entropy method to efficiently mask histology whole-slide images
Source: Sci Rep. 2023 Mar 15;13:4321. doi: 10.1038/s41598-023-29638-1 (PMC10017682; doi:10.1038/s41598-023-29638-1)
Supplement: Supplementary file 1 — Supplementary Information. [file 41598_2023_29638_MOESM1_ESM.pdf]

Supplemental Material

accompanying

An automatic entropy method to efficiently mask histology whole-slide images

Supplemental Figures

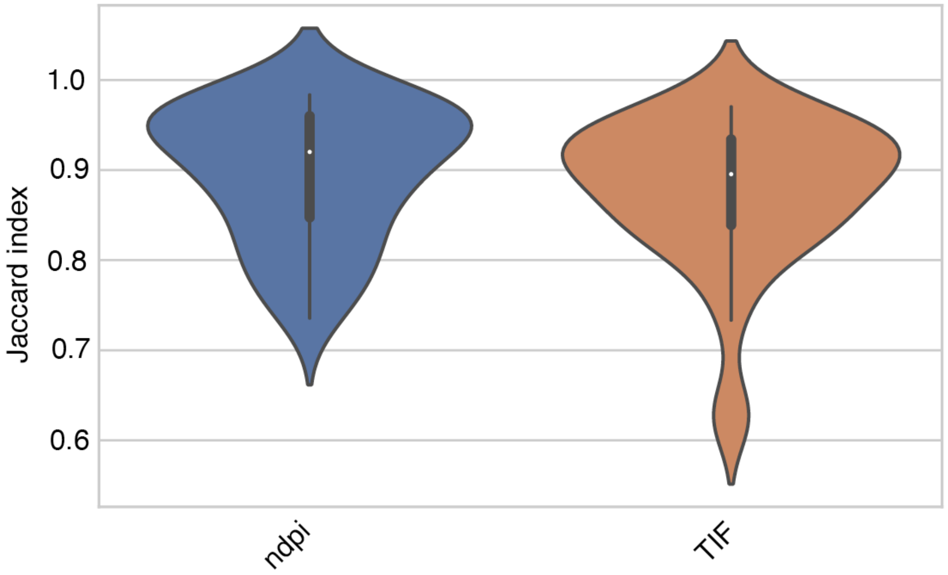

Supplemental Figure 1: EntropyMasker performance using different image formats (.ndpi or .TIF). Violin plot showing the effects of the two different image formats (.ndpi or .TIF) on `EntropyMasker` performance, as determined by the Jaccard index. n=33 images (.ndpi) and n=65 images (.TF) were analyzed as described in the **Methods**. Individual slide information is provided in **Supplemental Table 2**.

Supplemental Tables

Supplemental Table 1: Minimalistic baseline characteristics of the Athero-Express Biobank Study.

Baseline characteristics for the patients included in this study. Age: age in years at the time of inclusion. Note that we report the data for 56 patients. As explained in the Methods section under ‘Patient population’, plaque-material are considered ‘waste biomaterial’ and are allowed to be used without any personal information. In this study we also considered this ‘waste biomaterial’, hence no clinical information, e.g., age, is provided here nor when sharing data of the relevant samples (n=3).

**Variable**	**Description**	**Overall**
N		56
Age (mean ± s.d.)		69.5 (9.3)
Gender (N, %)	*female*	33.9 (19)
	male	66.1 (37)
Hospital (N, %)	*St. Antonius, Nieuwegein*	21.4 (12)
	UMC Utrecht	78.6 (44)
Artery type (N, %)	*carotid (left & right)*	85.7 (48)
	femoral/iliac (left, right or both sides)	12.5 (7)
	other arteries (renal, popliteal, vertebral)	1.8 (1)
Overall plaque phenotype	*atheromatous*	21.4 (12)
	fibroatheromatous	19.6 (11)
	fibrous	41.1 (23)

Supplemental Table 2: Slidenumbers as used for this project's algorithm design. Some slides were also used for figures. Stains used are macrophages (CD68), smooth muscle cells (SMCs, α-actin), neutrophils (CD66b), endothelial cells (CD34), red blood cells (glycophorin C, GLYCC), hematoxylin and eosin (HE) for nuclei, collagen (picrosirius red, SR), fibrin (FIBRIN), elastic Van Gieson (EvG). WSI are obtained at 40x magnification, and stored as z-stacked .TIF images as produced by the Roche Ventana iScan HT with 0.25 micron/pixel or .ndpi images as produced by the Hamamatsu C12000-22 Digital slide scanner with 0.23 micron/pixel.

**Slidenumber**	**Stain**	**Imagetype**	**Figure**
AE1299	CD34	TIF	
AE1354	CD34	TIF	
AE1675	CD34	TIF	
AE182	CD34	TIF	
AE2086	CD34	TIF	
AE2173	CD34	TIF	
AE2217	CD34	TIF	
AE2622	CD34	TIF	Figure 4 F
AE2637	CD34	TIF	
AE2742	CD34	TIF	
AE489	CD34	TIF	
AE839	CD34	TIF	
AE1	CD68	TIF	
AE100	CD68	TIF	Figure 4 E
AE100	CD68	TIF	
AE1001	CD68	TIF	
AE1006	CD68	TIF	
AE101	CD68	TIF	
AE1010	CD68	TIF	
AE1016	CD68	TIF	
AE1018	CD68	TIF	
AE102	CD68	TIF	

AE1299	EVG	TIF	
AE1354	EVG	TIF	
AE1675	EVG	TIF	
AE182	EVG	TIF	
AE2173	EVG	TIF	
AE2217	EVG	TIF	
AE2622	EVG	TIF	
AE2637	EVG	TIF	Figure 4 H
AE2742	EVG	TIF	
AE3151	EVG	TIF	
AE489	EVG	TIF	
AE839	EVG	TIF	
AE87	EVG	TIF	
AE1178	FIBRIN	TIF	
AE1299	FIBRIN	TIF	
AE146	FIBRIN	TIF	
AE1675	FIBRIN	TIF	
AE182	FIBRIN	TIF	
AE260	FIBRIN	TIF	
AE489	FIBRIN	TIF	Figure 4 D
AE87	FIBRIN	TIF	
AE99	FIBRIN	TIF	
AE3606	GLYCC	ndpi	
AE3756	GLYCC	ndpi	
AE3884	GLYCC	ndpi	Figure 4 B
AE4022	GLYCC	ndpi	
AE4270	GLYCC	ndpi	
AE4271	GLYCC	ndpi	
AE4272	GLYCC	ndpi	
AE4273	GLYCC	ndpi	
AE4274	GLYCC	ndpi	

AE4275	GLYCC	ndpi	
AE4276	GLYCC	ndpi	
AE4277	GLYCC	ndpi	
AE87	GLYCC	TIF	
AE1007	HE	ndpi	
AE1030	HE	ndpi	
AE1101	HE	ndpi	
AE1189	HE	ndpi	
AE1217	HE	ndpi	
AE1221	HE	ndpi	
AE1222	HE	ndpi	Figure 4 A
AE1227	HE	ndpi	
AE1229	HE	ndpi	
AE1230	HE	ndpi	
AE1237	HE	ndpi	
AE1238	HE	ndpi	
AE1381	HE	ndpi	
AE1382	HE	ndpi	
AE1466	HE	ndpi	
AE1468	HE	ndpi	
AE1473	HE	ndpi	Figure 3
AE1490	HE	ndpi	
AE1499	HE	ndpi	
AE1504	HE	ndpi	
AE1299	SMA	TIF	
AE1354	SMA	TIF	
AE1675	SMA	TIF	
AE2086	SMA	TIF	
AE2173	SMA	TIF	
AE2217	SMA	TIF	
AE2622	SMA	TIF	Figure 4 G

AE2637	SMA	TIF	
AE2742	SMA	TIF	
AE3151	SMA	TIF	
AE489	SMA	ndpi	
AE839	SMA	TIF	
AE1299	SR	TIF	
AE1354	SR	TIF	Figure 4 C
AE182	SR	TIF	
AE2086	SR	TIF	
AE2217	SR	TIF	
AE2622	SR	TIF	
AE2637	SR	TIF	
AE2742	SR	TIF	
AE489	SR	TIF	

Supplemental Table 3: Mean values for different staining methods and 4 different evaluation metrics. Stains used are: macrophages (CD68, n=9), smooth muscle cells (SMCs, α-actin, n=12), endothelial cells (CD34, n=12), red blood cells (glycophorin C, GLYCC,n =13), hematoxylin and eosin (HE, n=20) for nuclei, collagen (picrosirius red, SR, n=9), fibrin (FIBRIN, n=9), elastic Van Gieson (EvG, n=13).

Stain	Method	mean Jaccard index	mean Sensitivity	mean False positive rate	mean Pixel accuracy
HE	EntropyMasker	0.94	0.99	0.0155	0.99
GLYCC		0.83	0.88	0.0083	0.98
FIBRIN		0.89	1.00	0.0132	0.99
SR		0.90	1.00	0.0214	0.98
SMA		0.91	0.97	0.0146	0.98
EVG		0.83	0.97	0.0313	0.97

CD68		0.82	0.96	0.0205	0.98
CD34		0.92	0.98	0.0192	0.98
HE	Otsu	0.79	0.80	0.0024	0.94
GLYCC		0.31	0.31	0.0003	0.89
FIBRIN		0.62	0.62	0.0073	0.95
SR		0.59	0.59	0.0014	0.94
SMA		0.32	0.33	0.0936	0.78
EVG		0.55	0.58	0.0046	0.91
CD68		0.47	0.50	0.0697	0.86
CD34		0.60	0.60	0.0015	0.92
HE	slideMask	0.81	0.81	0.0001	0.95
GLYCC		0.43	0.43	0.0035	0.91
FIBRIN		0.49	0.49	0.0012	0.94
SR		0.65	0.65	0.0010	0.95
SMA		0.17	0.17	0.0014	0.84
EVG		0.55	0.56	0.0033	0.92
CD68		0.28	0.28	0.0037	0.91
CD34		0.61	0.82	0.1722	0.84
HE	adaptive_mean	0.87	0.88	0.0036	0.97
GLYCC		0.65	0.71	0.0363	0.92
FIBRIN		0.85	0.89	0.0046	0.98
SR		0.73	0.75	0.0148	0.96
SMA		0.65	0.70	0.0248	0.93
EVG		0.71	0.75	0.0152	0.95
CD68		0.66	0.62	0.0132	0.96

CD34		0.59	0.75	0.1722	0.84
HE	adaptive_gaussian	0.92	0.94	0.0045	0.98
GLYCC		0.66	0.82	0.0479	0.93
FIBRIN		0.90	0.96	0.0070	0.99
SR		0.81	0.87	0.0173	0.97
SMA		0.78	0.83	0.0099	0.96
EVG		0.68	0.75	0.0448	0.93
CD68		0.70	0.76	0.0166	0.97
CD34		0.54	0.93	0.2660	0.79

Supplemental Table 4: Quantitative results of different masking methods for both high and relatively low-resolution images. The high resolution WSIs are at 20x magnification and low resolution WSIs are at 5x magnification. WSIs at 20x magnification were stored digitally as z-stacked .TIF (Roche) at 0.25 micron/pixel or .ndpi (Hamamatsu) at 0.23 micron/pixel brightfield microscopy images. Average Jaccard Index for each method and in each resolution is shown in bold.

**image name**	**Method**	**Jaccard Index**	**resolution**
AE1203.UMC.GLYCC.20150306	EntropyMasker	0.869	high
AE2633.EVG.910	EntropyMasker	0.791	high
AE1468.GLYCC	EntropyMasker	0.945	high
AE2633.HE	EntropyMasker	0.767	high
AE1203.CD68	EntropyMasker	0.954	high
AE2633.CD68.911	EntropyMasker	0.828	high
AE2937.HE	EntropyMasker	0.490	high
AE2937.CD68	EntropyMasker	0.603	high
AE2937.SMA	EntropyMasker	0.701	high
AE2937.EVG	EntropyMasker	0.528	high
AE2633.SMA	EntropyMasker	0.795	high

**Average EntropyMasker**		**0.752**	high
AE1203.UMC.GLYCC.20150306	slidemask	0.007	high
AE2633.EVG.910	slidemask	0.075	high
AE1468.GLYCC	slidemask	0.460	high
AE2633.HE	slidemask	0.020	high
AE1203.CD68	slidemask	0.052	high
AE2633.CD68.911	slidemask	0.001	high
AE2937.HE	slidemask	0.453	high
AE2937.CD68	slidemask	0.031	high
AE2937.SMA	slidemask	0.076	high
AE2937.EVG	slidemask	0.512	high
AE2633.SMA	slidemask	0.065	high
**Average slidemask**		**0.159**	high
AE1203.UMC.GLYCC.20150306	otsu	0.002	high
AE2633.EVG.910	otsu	0.000	high
AE1468.GLYCC	otsu	0.667	high
AE2633.HE	otsu	0.516	high
AE1203.CD68	otsu	0.070	high
AE2633.CD68.911	otsu	0.059	high
AE2937.HE	otsu	0.407	high
AE2937.CD68	otsu	0.201	high
AE2937.SMA	otsu	0.043	high
AE2937.EVG	otsu	0.455	high
AE2633.SMA	otsu	0.061	high
**Average otsu**		**0.226**	high
AE1203.UMC.GLYCC.20150306	adaptive_mean	0.578	high
AE2633.EVG.910	adaptive_mean	0.490	high
AE1468.GLYCC	adaptive_mean	0.738	high

AE2633.HE	adaptive_mean	0.539	high
AE1203.CD68	adaptive_mean	0.706	high
AE2633.CD68.911	adaptive_mean	0.599	high
AE2937.HE	adaptive_mean	0.406	high
AE2937.CD68	adaptive_mean	0.497	high
AE2937.SMA	adaptive_mean	0.497	high
AE2937.EVG	adaptive_mean	0.379	high
AE2633.SMA	adaptive_mean	0.511	high
**Average adaptive_mean**		**0.540**	high
AE1203.UMC.GLYCC.20150306	adaptive_gaussian	0.686	high
AE2633.EVG.910	adaptive_gaussian	0.567	high
AE1468.GLYCC	adaptive_gaussian	0.820	high
AE2633.HE	adaptive_gaussian	0.617	high
AE1203.CD68	adaptive_gaussian	0.807	high
AE2633.CD68.911	adaptive_gaussian	0.658	high
AE2937.HE	adaptive_gaussian	0.449	high
AE2937.CD68	adaptive_gaussian	0.549	high
AE2937.SMA	adaptive_gaussian	0.590	high
AE2937.EVG	adaptive_gaussian	0.435	high
AE2633.SMA	adaptive_gaussian	0.605	high
**Average adaptive_gaussian**		**0.617**	high
AE1499.UMC	EntropyMasker	0.968	low
AE1230.UMC	EntropyMasker	0.890	low
AE1237.HE_x20_z0	EntropyMasker	0.962	low
AE1473.UMC	EntropyMasker	0.922	low
AE1382.HE_x20_z0	EntropyMasker	0.975	low
AE2633.HE.macro	EntropyMasker	0.954	low
AE2633.SMA.912.macro	EntropyMasker	0.822	low
AE1203.CD68.macro	EntropyMasker	0.947	low

AE2937.HE.macro	EntropyMasker	0.567	low
AE1203.CD34.macro	EntropyMasker	0.906	low
AE2633.CD68.911.macro	EntropyMasker	0.847	low
**Average EntropyMasker**		**0.887**	**low**
AE1499.UMC	slidemask	0.897	low
AE1230.UMC	slidemask	0.628	low
AE1237.HE_x20_z0	slidemask	0.219	low
AE1473.UMC	slidemask	0.868	low
AE1382.HE_x20_z0	slidemask	0.946	low
AE2633.HE.macro	slidemask	0.028	low
AE2633.SMA.912.macro	slidemask	0.099	low
AE1203.CD68.macro	slidemask	0.058	low
AE2937.HE.macro	slidemask	0.573	low
AE1203.CD34.macro	slidemask	0.858	low
AE2633.CD68.911.macro	slidemask	0.001	low
**Average slidemask**		**0.470**	**low**
AE1499.UMC	otsu	0.785	low
AE1230.UMC	otsu	0.947	low
AE1237.HE_x20_z0	otsu	0.716	low
AE1473.UMC	otsu	0.647	low
AE1382.HE_x20_z0	otsu	0.911	low
AE2633.HE.macro	otsu	0.783	low
AE2633.SMA.912.macro	otsu	0.153	low
AE1203.CD68.macro	otsu	0.586	low
AE2937.HE.macro	otsu	0.551	low
AE1203.CD34.macro	otsu	0.850	low
AE2633.CD68.911.macro	otsu	0.670	low
**Average otsu**		**0.691**	**low**

AE1499.UMC	adaptive_mean	0.883	low
AE1230.UMC	adaptive_mean	0.807	low
AE1237.HE_x20_z0	adaptive_mean	0.894	low
AE1473.UMC	adaptive_mean	0.834	low
AE1382.HE_x20_z0	adaptive_mean	0.877	low
AE2633.HE.macro	adaptive_mean	0.902	low
AE2633.SMA.912.macro	adaptive_mean	0.757	low
AE1203.CD68.macro	adaptive_mean	0.859	low
AE2937.HE.macro	adaptive_mean	0.576	low
AE1203.CD34.macro	adaptive_mean	0.880	low
AE2633.CD68.911.macro	adaptive_mean	0.784	low
**Average adaptive_mean**		**0.823**	**low**
AE1499.UMC	adaptive_gaussian	0.932	low
AE1230.UMC	adaptive_gaussian	0.853	low
AE1237.HE_x20_z0	adaptive_gaussian	0.927	low
AE1473.UMC	adaptive_gaussian	0.887	low
AE1382.HE_x20_z0	adaptive_gaussian	0.926	low
AE2633.HE.macro	adaptive_gaussian	0.834	low
AE2633.SMA.912.macro	adaptive_gaussian	0.779	low
AE1203.CD68.macro	adaptive_gaussian	0.904	low
AE2937.HE.macro	adaptive_gaussian	0.592	low
AE1203.CD34.macro	adaptive_gaussian	0.921	low
AE2633.CD68.911.macro	adaptive_gaussian	0.792	low
**Average adaptive_gaussian**		**0.850**	**low**
